# Cognitive health begins at conception: addressing dementia as a lifelong and preventable condition

**DOI:** 10.1186/1741-7015-11-246

**Published:** 2013-11-19

**Authors:** Jennifer H Barnett, Vladimir Hachinski, Andrew D Blackwell

**Affiliations:** 1 Department of Psychiatry, University of Cambridge, Cambridge Biomedical Campus, Cambridge, CB2 2QQ, UK; 2 Cambridge Cognition Ltd, Tunbridge Court, Tunbridge Lane, Bottisham, CB25 9TU, UK; 3 Department of Clinical Neurological Sciences, London Health Sciences Centre, University of Western Ontario, University Hospital, 339 Windermere Road, London, Ontario, N6A 5A5, Canada

**Keywords:** Dementia, Alzheimer’s disease, Prevention, Epidemiology

## Abstract

**Background:**

Dementia is a major public health problem that poses an increasing burden on the health and wealth of societies worldwide. Because the efficacy of current treatments is limited, increasing efforts are required to prevent the diseases that cause dementia.

**Discussion:**

We consider the evidence that lifelong prevention strategies may be an effective way to tackle the national burden of dementia in the absence of a cure. The links between lifestyle and cardiovascular disease are widely understood and accepted, but health professionals and patients remain unconvinced about the extent to which risk for dementia can be modified. However, there is strong evidence that at least half of risk for dementia is attributable to lifestyle factors such as diet, exercise and smoking. Moreover, the disease processes that result in dementia develop over several decades, implying that attempts to ameliorate them need to start early in life. Some modifiable risk factors for dementia act from the earliest stages of life, including in utero.

**Summary:**

Rebalancing efforts from the development of treatments to increased emphasis on prevention may be an alternative means to reducing the impact of dementia on society.

## Background

Worldwide, 36 million people suffer from dementia, costing an annual US$600 billion [1]. Dementia is a clinical state due to one of several underlying pathologies, most commonly Alzheimer’s disease (AD) or cerebrovascular disease, with more rare causes including dementia with Lewy bodies and frontotemporal dementia [2]. Conclusively diagnosing which of these diseases is present in an individual is only possible post mortem, so in clinical practice, patients are ascribed a probable differential diagnosis and then treated accordingly. In reality, both post mortem [3-5] and brain imaging studies [6,7] show that many, and perhaps most, patients diagnosed with either Alzheimer’s or vascular dementias are in fact experiencing the cumulative effects of both pathologies. Moreover, cerebrovascular disease appears to act synergistically with Alzheimer’s pathology to worsen the cognitive performance of the patient [3,8], lowering the threshold at which the symptoms of dementia emerge.

To an individual patient, accurate differential diagnosis matters only to the extent that it provides the most accurate prognosis, and triggers the most effective treatment course. Unfortunately, at present there are no licensed treatments for vascular dementia, and available AD treatments bring only short-term relief, typically slowing the worsening of symptoms for 6 to 12 months. Recent efforts to develop new drugs to treat symptoms, or to slow disease progress, have been disappointing, and after some high-profile failures [9-11] there is a growing concern that the pharmaceutical industry may reduce investment in the field. If effective and affordable pharmacological or biological treatments do not emerge, preventing the diseases that cause dementia will become ever more important.

While the links between lifestyle and cardiovascular disease are widely accepted, considerable fatalism exists among both health professionals and patients about the extent to which risk for dementia can be modified. Consequently, it is timely to recall that around half of risk for dementia is attributable to lifestyle factors such as diet, exercise and smoking and that this risk is accrued throughout life, so attempts to ameliorate risk need to start early. Indeed there is growing evidence that modifiable risk factors for dementia act from the earliest stages of life, including in utero.

## Discussion

### Dementia is largely preventable

Age is the biggest risk factor for dementia, with risk for both AD and vascular dementia (VaD) doubling every 5 years over 65 [1,12]. Late-onset AD and VaD are both moderately heritable and although most individual genes increase risk by only a small amount, the apoliprotein E (APOE) ϵ4 allele confers a threefold to four fold increase in risk for AD [13] and a more modest increase in VaD [14]. Men are at higher risk for VaD, and women for AD, alongside people with a history of head injury.

**Figure 1:**
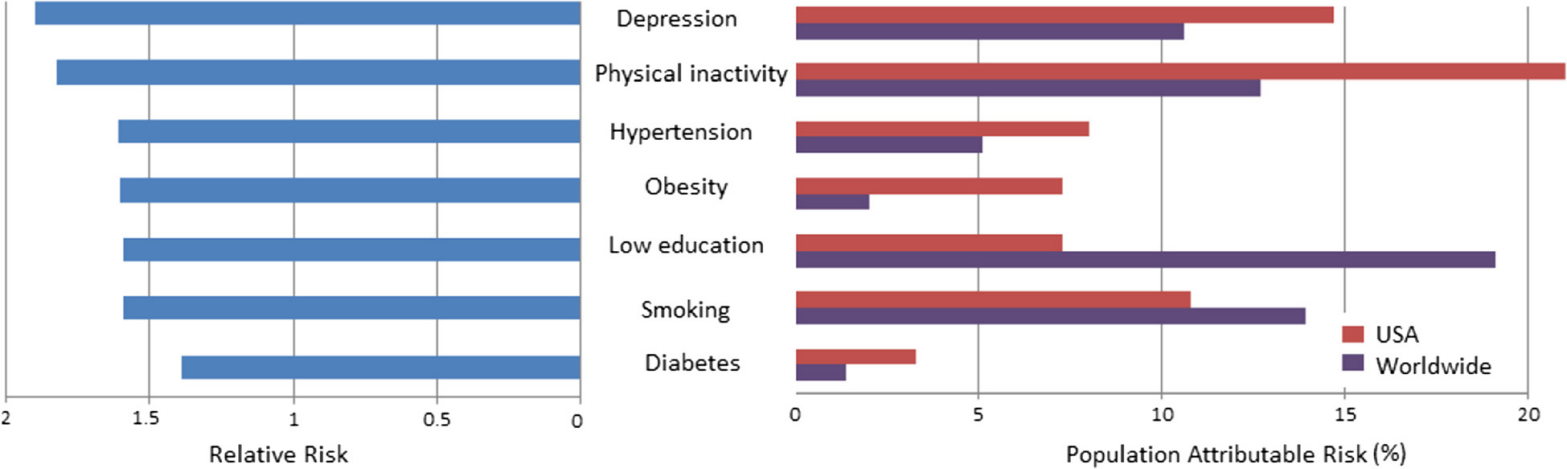
**Relative and population attributable risk estimates for seven modifiable risk factors for Alzheimer’s disease (data from Barnes and Yaffe**[16]**).**

Against this background of non-modifiable risk, many preventable factors considerably exacerbate an individual’s risk (see Figure 1). Risk factors for VaD are those associated with general cardiovascular health, and three-quarters of patients with VaD and more than half of those with vascular cognitive impairment have a history of stroke, compared with only 5% to 7% of people with AD [15]. Half of AD risk is explained by seven lifestyle-related factors: diabetes, hypertension, obesity, smoking, depression, cognitive activity/education and physical activity [16]. Evidence is particularly strong for a cluster of metabolic factors including hypertension, serum lipids, diabetes, and obesity, and exposure to these factors during midlife (as opposed to late life) seems particularly predictive of later dementia risk [17-20].

Importantly, the protective effects of healthy lifestyles are as great for dementia as for other late-life diseases. Prospective cohort studies show that individuals who adhere to a Mediterranean-style diet experience a greater reduction in risk for diseases such as dementia, Parkinson’s and stroke (overall RR = 0.87 (95% CI 0.81 to 0.94)) than the reduction in risk seen for cardiovascular disease (RR = 0.90 (95% CI 0.87 to 0.93)), cancer (RR = 0.94, (95% CI 0.92 to 0.96)), or overall mortality (RR = 0.92 (95% CI 0.90 to 0.94) [21]). Similarly, meta-analyses of the effects of physical exercise suggest that it can reduce risk for AD by 45% (RR 0.55 (95% CI 0.36 to 0.84) [22]), a protective effect comparable to that achieved against type II diabetes (RR 0.63 (95% CI 0.49 to 0.79) [23]), or coronary heart disease (RR 0.73 (95% CI 0.66 to 0.80) [24]). The effects of reducing modifiable risk may be particularly dramatic in individuals at high non-modifiable risk. For example, in the Honolulu study, relative risk for impaired cognition was 13 among individuals with both the APOE ϵ4 allele and hypertension, but was reduced to just 2 by effective hypertension treatment [25]. Similarly, the amyloid deposition associated with APOE ϵ4 carrier status has recently been shown to be exacerbated by hypertension and reduced by treatment to control blood pressure [26]. Those who are at highest *a priori* risk for dementia are, by the same token, most likely to benefit from strategies to reduce modifiable risk factors.

A major barrier to implementing effective prevention strategies for dementia is that the risk-modifying behaviors identified in observational studies have rarely been replicated in randomized clinical trials. Secondary prevention for VaD, for example using angiotensin-converting enzyme (ACE) inhibitors, can significantly prevent further strokes and subsequent cognitive decline [27]. However, studies aiming to prevent dementia among the general elderly population, or to prevent worsening of symptoms among individuals with dementia, have mostly failed. These include trials of non-steroidal anti-inflammatory drugs (NSAIDs) [28] and vitamin E [29], and interventions for low folate and vitamin B12 [30]. This pattern of failure to turn promising observations into successful interventions may reflect the effect on observational studies of reverse causality; for example, individuals experiencing the early stages of cognitive decline are probably less likely to eat well and take part in physical activity. Associations between risk factors and dementia may also be confounded by other behavioral factors, personality, and comorbid conditions. One particularly plausible explanation is that the failure of these trials simply means that attempts to prevent dementia need to start earlier, and last longer, than any so far conducted.

### Dementia risk begins at birth

Both AD and VaD are the result of disease processes which usually develop over several decades. While the evidence for association between lifestyle factors and dementia risk is strongest for exposure in midlife, lifestyles in middle age usually reflect lifelong patterns of behavior, so individuals who eat well and exercise in midlife are likely to be benefiting from the cognitively protective effects of a lifetime of such behaviors. For many adult diseases, substantial risk can be traced to early childhood and, in some cases, back to the womb. The ‘Barker hypothesis’ that suboptimal prenatal and early life environment increases risk for adult disease, has been convincingly demonstrated for conditions including stroke, heart disease, insulin resistance, and hypertension [31]. Although considerably less research has addressed this, there are a number of ways in which this is probably also true for dementia. Neonatal environment, particularly diet, can have a major impact on the development of cognitive function. Breastfeeding confers an IQ advantage [32], and in vulnerable groups such as premature babies, optimal nutrition in the first few weeks can improve cognitive and brain development throughout childhood and adolescence [33], and low birthweight babies show poorer cognitive development, and poorer cognitive function even as adults [34].

General indices of early development, such as limb length [35] are also associated with risk for dementia. People who have no symptoms of dementia despite significant AD pathology on autopsy tend to have larger brains and a greater number of neurons than those with no AD pathology [36]. Optimal physical growth in the brain thus seems to confer some resilience to the effects of AD pathology and neurodegeneration.

Some environmental effects may act directly on the developing brain, for example through prenatal exposure to toxins. Routes that are more indirect are also likely: for example, maternal smoking may increase later risk for dementia by influencing lifelong cardiovascular and metabolic health [37]. Similarly, an individual who is obese in midlife (with a body mass index ≥30) has an approximately doubled risk for later AD, while an individual who is overweight (body mass index (BMI) between 25 and 30) has a 35% increased risk [38]. But midlife obesity is itself significantly driven by early life: both high and low birthweight babies are at increased risk for later obesity [39].

Lifetime associations between cognition and health may also operate through behavioral choices. In a New Zealand birth cohort, childhood self-control, an executive function dependent on prefrontal cortical integrity, predicted a range of adult physical health outcomes [40] including metabolic status, periodontal disease, and drug and tobacco dependence. Some of the relationship between self-control and later health was explained by the approximately doubled likelihood of children with poor self-control experiencing common adolescent ‘snares’, such as starting smoking at an early age.

### Optimizing the cognitive health of individuals and societies

Throughout life, many potentially treatable or reversible causes of cognitive impairment can occur. Among children, disorders such as attention deficit hyperactivity disorder (ADHD) and dyslexia impair specific cognitive functions that can considerably impact a child’s educational attainment. Among adults, reversible causes of cognitive impairment can include stress, sleep deprivation, drug and alcohol use, depression, thyroid disease, and vitamin deficiencies. If not detected, these conditions may have long term consequences for cognitive health, including structural or functional brain changes that may be minimized by early treatment.

Individuals with greater educational and occupational attainment, those with higher IQ, and those who undertake complex mental activity later in life are all at lower risk of dementia, and show slower cognitive decline after a diagnosis of dementia [41]. The idea that dementia can be prevented through interventions such as ‘brain training’ programs has captured the public imagination. It is estimated that consumers spent around US$500 million on such products in 2012 [42] though little evidence supports their benefits among healthy elderly individuals.

Functioning at a suboptimal cognitive level at any point in the lifespan may reduce engagement in activities which protect brain health, including education, employment, and healthy lifestyles. A child with undetected dyslexia is unlikely to undertake higher education, a young adult with poor concentration is unlikely to rise through the career ranks, and an older person with a depression-induced memory complaint is unlikely to take part in cognitively challenging activities. To maximize the cognitive health of the population it is therefore important that cognitive function is monitored throughout life, so that any changes can be detected and, where appropriate, treated.

### Turning epidemiology into prevention

**Figure 2:**
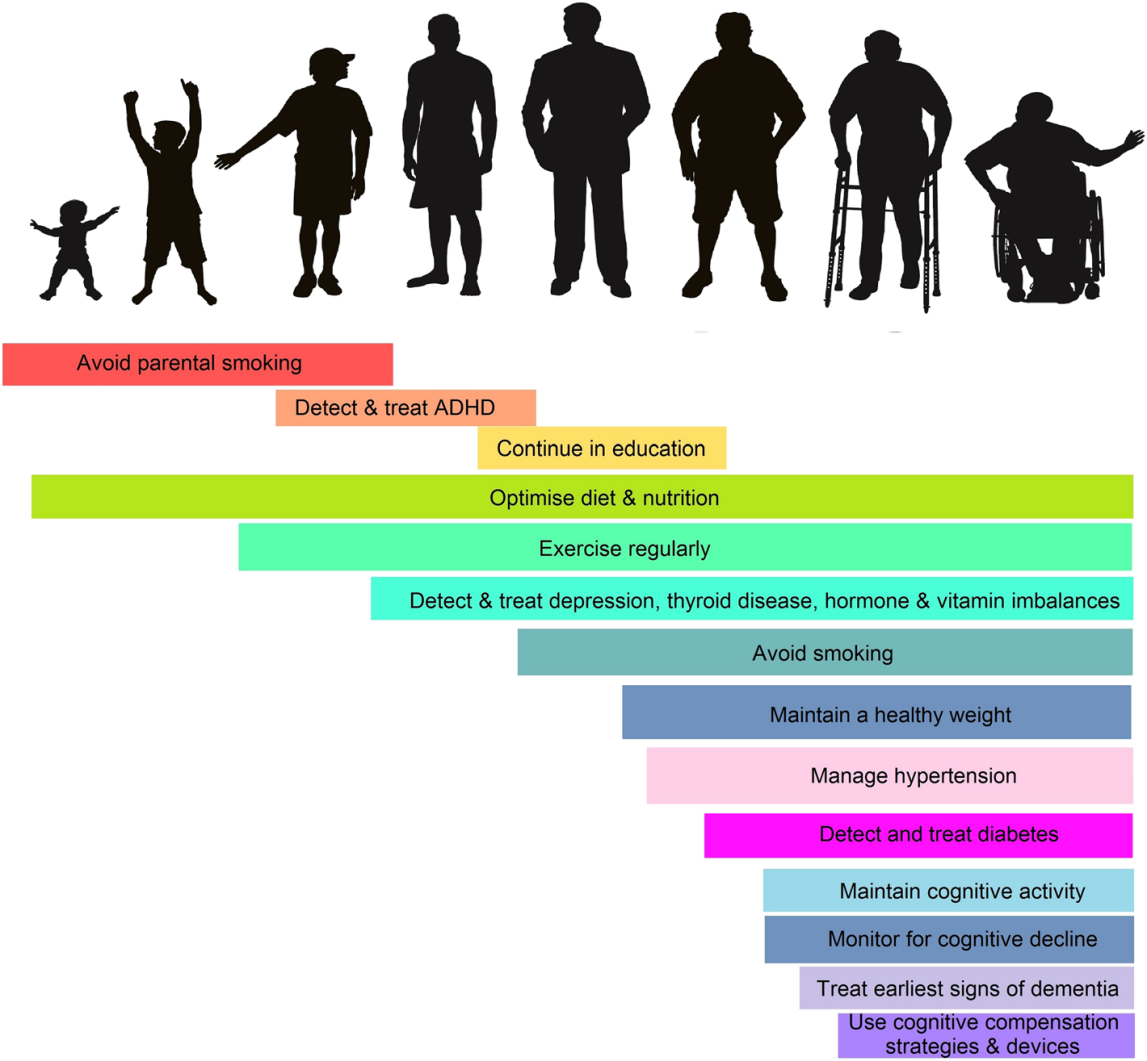
Preventing dementia throughout life.

The epidemiological evidence described above provides strong evidence of associations between lifestyle factors and risk for dementia but these associations do not prove causality. Nonetheless, the existence of these associations does suggest that some risk for dementia could be reduced through the protection of cognitive health throughout the lifespan (Figure 2). Strategies might include the optimizing of diet through neonatal periods as well as later in life, further reducing behaviors such as smoking, which may be directly or indirectly harmful to cognitive health, and the early detection and treatment of reversible causes of cognitive impairment throughout life.

Public policy impacts dementia risk in a number of ways. Many countries now have national dementia strategies and aim to increase research capacity as well as increasing public awareness. Given the potential burden of dementia on society, it should be a public health priority to educate both clinicians and the population to understand that risk for dementia is driven by lifestyle choices as well as genetic predisposition. Using an internet-based survey, we recently asked more than 1,000 UK general practitioners (GPs) whether a person could alter their risk of developing dementia. While a majority (59%) agreed they could, 32% weren’t sure and 10% thought they could not; this is concerning, since both the scientific literature and current clinical practice guidelines [2] unequivocally support the existence of modifiable factors in contributing to dementia risk. Increased training in dementia may be a useful first step: in a separate study, half of English GPs felt they had not received sufficient training to help them diagnose and manage dementia [43].

The modifiable factors that most predict risk for dementia are conditions (obesity, hypertension, diabetes), or lifestyle factors (diet, cognitive and physical activity, smoking cessation) where a GP is best placed to effect change. However moving beyond demonstrating associations to demonstrating causality, efficacy, and ultimately, cost effectiveness is something that only randomized controlled trials can tackle. While small-scale projects do exist in the UK, we lag behind much of Europe where several large-scale prevention initiatives are occurring [44]. These aim to apply the methodological rigor of a randomized controlled trial to multimodal lifestyle interventions among older adults who are not yet demented. They are characterized by large sample sizes, long follow-ups, and an intervention that is typically carried out by nurses or allied health professions, but are limited in that they are intervening only in late life, which may already be too late.

## Summary

In recent years, the scientific understanding of dementia has shifted from that of a late-life disease that cannot be prevented to that of a lifelong disease process, where factors such as diet and education impact risk from the earliest stages of life. As yet, there is little evidence from large-scale studies about the efficacy of individual or combined lifestyle interventions in preventing dementia, but in the absence of disease-modifying therapies, the prevention of dementia should be prioritized by clinicians and healthcare policy, and by more ambitious research efforts. In particular, the public health message that cognitive decline and dementia are preventable should be reinforced among both clinicians and patients.

## Abbreviations

ACE: Angiotensin-converting enzyme; AD: Alzheimer’s disease; ADHD: Attention deficit hyperactivity disorder; APOE: Apolipoprotein E; BMI: Body mass index; CI: Confidence interval; GP: General practitioner; IQ: Intelligence quotient; NSAID: Non-steroidal anti-inflammatory drug; RR: Relative risk; VaD: Vascular dementia.

## Competing interests

JHB and ADB are employees of, and own shares in, Cambridge Cognition. VH declares no competing interests.

## Authors’ contributions

ADB and JHB conceived the basis of the article, based in part on discussions with VH who subsequently contributed additional ideas and critical input. JHB drafted the initial manuscript and figures, incorporating contributions from all authors. All authors read and approved the final manuscript.
